# A Ridesharing Choice Behavioral Equilibrium Model with Users of Heterogeneous Values of Time

**DOI:** 10.3390/ijerph18031197

**Published:** 2021-01-29

**Authors:** Xingyuan Li, Jing Bai

**Affiliations:** 1 School of Business, East China University of Science and Technology, Shanghai 200237, China; 2 School of Economics and Management, Yanshan University, Qinhuangdao 066004, China; bjr@stumail.ysu.edu.cn

**Keywords:** ridesharing, heterogeneous values of time, traffic assignment, multiclass users

## Abstract

Travelers decide whether to participate in ridesharing based on the trade-off between the travel time and the expense. However, it is still unclear how travelers’ values of time affect their ridesharing behaviors on the congested network. To this end, a path-based ridesharing traffic assignment model was proposed by considering travelers’ heterogenous values of time. In the proposed model, travelers are divided into several classes according to their values of time, and travelers in each class choose their travel modes and routes simultaneously which cost the least. Moreover, travelers in different classes could share the same vehicle to complete their trips together in the proposed model. This paper further discusses how the high-occupancy toll lane affects travelers’ ridesharing behaviors. Numerical results show that: (1) travelers with different values of time show differences in their ridesharing behavior; (2) the single-class ridesharing traffic assignment model may miscalculate the ridesharing scale of users; and (3) building high-occupancy toll lanes plays a positive role in promoting ridesharing for travelers with heterogeneous values of time.

## 1. Introduction

Urban traffic congestion, automobile exhaust pollution, and energy shortages have become common urban diseases. In the United States, the cost of congestion alone has nearly quadrupled from $41 billions in 1982 to $153 billions in 2013, which includes sixfold growth in wasted fuel and nearly tripled growth in travel time delay [1]. Transportation accounted for 27% of the total greenhouse gas emissions of the United States in 2013 and increased more from 1990 to 2013 in absolute terms than any other sector [2]. In China, the CO_2_ emissions of Shanghai have also increased greatly during these years, and mode share plays a significant role in CO_2_ emissions in the urban transport sector [3]. In recent years, due to the prosperity of sharing economic, a new breakthrough has been found to deal with above tricky issues. The shared mobility, one of the most important business models in the sharing economic, has many forms, including carsharing, ridesourcing, carpooling, taxi-sharing, bikesharing, scooter sharing, and ridesharing [4]. Among these shared mobility services, ridesharing in particular can substantially reduce the number of single-occupancy vehicles on roads, which may ameliorate the perennial traffic congestion, as well as reduce energy consumption [5,6,7]. It could also meet the increasing demand for mobility without adding to, or even curbing, the environmental footprint of the transportation sector by utilizing the empty spaces in traveling vehicles, rather than recruiting drivers whose sole purpose is to transport passengers [8]. Ridesharing is a travel mode that can join trips of at least two participants who intend to reduce their travel cost by sharing a vehicle [8,9]. Although it has been practiced and studied for many years, ridesharing’s modal share has been declining in the past years, due to the travelers’ behavioral barriers and the technical obstacle [9]. However, with the prevalence of smartphones enhanced by communications technology and GPS and the advanced engineering technologies, such as cloud computing, and operation management technique, the on-demand requests for ridesharing get quick responses by the matching agencies in recent years, which enable the participants to change their itineraries expediently. Accordingly, ridesharing gradually becomes a popular travel mode again. Many ridesharing platforms operated by commercial companies have been rapidly growing, such as Uber, DiDi, and Grab [10]. In addition to the social benefits mentioned above, there are many benefits to ridesharing at the individual level. For example, ridesharing participants could reduce their travel costs by sharing rides, save travel times by employing high-occupancy vehicle (HOV) lanes, or have preferential parking [8].

It is worth studying how to motivate travelers to participate in ridesharing due to its plentiful benefits. In the context of ridesharing, travelers have three travel modes, i.e., solo drivers (SDs), ridesharing drivers (RDs), and ridesharing passengers (RPs). Those who choose to be RDs provide rides for RPs who have the similar itineraries as the RDs, and those who choose to be RPs pay fees to the matched RDs by a transportation network company [11,12]. Nevertheless, whether travelers decide to share/take rides depends on the trade-off between the travel time and the expense of their trips, since one may experience different travel cost on the same route if he or she chooses different travel modes. Accordingly, travelers’ values of time (VOTs) have a significant influence on their ridesharing decisions. It is well-known that travelers are heterogeneous with their VOTs due to their socioeconomic characteristics and trip purpose [13,14], and travelers’ VOTs might vary over time [15,16]. For example, the VOTs on the way to work vary according to the departure time for commuters, thus commuters may adjust their departure times to avoid the risk of being late [15]. Although the relation between travelers’ ridesharing behaviors and traffic congestion has been studied with the assumption of homogenous travelers [11,12,17,18,19,20], it is still unclear how travelers’ VOTs affect their ridesharing behaviors.

The purpose of this paper is to explore how travelers will behave in ridesharing when their VOTs are heterogeneous. A path-based ridesharing traffic assignment model is proposed by considering travelers’ heterogenous VOTs. A more realistic ride-matching constraint is proposed to reflect the fact that travelers with different VOTs could share a same vehicle. The solution of the proposed model was proved to exist. This research was embedded only in a quantitative tradition of research. The formulation is extended by considering the presence of high-occupancy toll (HOT) lanes and explored how HOT lanes would impact the travelers’ ridesharing behaviors. By conducting numerical experiments, the results show that travelers with lower values of time are sensitive to driving cost, while those with higher values of time are sensitive to inconvenience cost. Besides, travelers’ willingness of ridesharing might decline as their values of time increase, which indicates that the single-class ridesharing traffic assignment model may miscalculate the scale of users’ participation of ridesharing. Finally, building HOT lanes plays a positive role to encourage travelers to participate in ridesharing activities

The remainder of this paper is arranged as follows. Section 2 reviews relevant literature. Section 3 formulates a traffic assignment model of multi-class users with ridesharing. Section 4 gives the equivalent variational inequality and existence of the model solution. Numerical results are analyzed in Section 5. The last section concludes the paper.

## 2. Literature Review

Research on ridesharing has developed rapidly in recent years. The major studies focus on ridesharing systems. The questions for this type of research include ride-matching and routing optimization algorithm design, pricing strategy design, and regulation policies for ridesharing market. Studies about these problem usually assume that the travel times are known or estimated and the congestion is not considered [21,22]. Agatz et al. [23] considered the matching problem of drivers and riders with the aim of minimizing the total system-wide vehicle miles and individual costs and developed optimization-based approaches. Stiglic et al. [7] found that small increases in flexibility can result in a significant increase in matching rate, especially in systems with low participation rates. Masoud et al. [24] devised a decomposition algorithm to solve the many-to-many matching problem. Long et al. [21] proposed a ride-sharing model which maximizes both the total generalized trip cost saving and the number of matches to investigate the effect of the travel time uncertainty on ridesharing. The results show that a ridesharing match based on deterministic travel time may be infeasible in a stochastic ride-sharing system. Peng et al. [25] devised a payment for ride-sharing and proposed a stable matching model which aims to minimize the travel cost of all commuters. They found that the compensation, time window and driver-to-rider ratios affect the matching rate. Gambella et al. [22] integrated an optimization module into a dynamic real-time decision support system for ridesharing at city-scale, where the information is received by the Internet of Thing (IoT) infrastructure. The simulation results show that making ridesharing aware of IoT data has a positive impact on the number of users served and vehicle utilization. Besides, Benjaafar et al. [26] provided insights into how product-sharing platforms may affect an individual’s decision to own. Castillo et al. [27] explored the potential benefits of state-contingent pricing when demand for rides is stochastic. Taylor [28] also explored how stochasticity in market conditions may affect the platform’s pricing and compensation decisions. Bimpikis et al. [10] explored spatial price discrimination in the context of a ride-sharing platform that serves a network of locations.

Another research topic is to investigate how ridesharing affects traffic congestion and how transportation planners should make decisions from the perspective of overall transportation system optimization [11,12,17,18,19,20]. Studies about these problems assume that travel times are endogenized by travelers’ travel choice (e.g., route choice and mode choice), and models about these ridesharing problems are usually formulated as traffic assignment problems or equilibrium problems [21]. The notion of “user equilibrium” was first proposed by Wardrop [29], which is the basic principle of traffic assignment. Beckmann et al. [30] then formulated an equivalent nonlinear optimization model of UE conditions with monotone link performance functions. Many traffic assignment models and effective algorithms under diverse scenarios have been proposed subsequently and gradually developed into a steam of research in the transportation literature [31,32]. Daganzo [33] incorporated carpooling into the network equilibrium and studied the issue of travelers’ route choice behaviors by charging differential toll to high-occupancy vehicles and single-occupancy vehicles. Yang and Huang [34] dealt with carpooling behavior and congestion pricing with or without HOV lanes in a multilane highway. They found toll differentiation made available for special cases and derived the uniform tolls for a second-best social optimum with HOV lanes. Xu et al. [35] studied the relation between ridesharing activities and traffic congestion in the network by proposing a mathematical programming model. The computational results show that the utilization of ridesharing increases as the congestion increases. Xu et al.’s [35] model assumes that ridesharing occurs in same origin–destination (OD) pair and the vehicle capacity was not taken into account. To relax these assumptions, Xu et al. [17] further proposed a mixed complementarity model and made ridesharing happen on different OD pairs. A side constraint was added to reflect the ride-matching relationships between ridesharing drivers and passengers. However, in Xu et al.’s [17] model, passengers had to transfer to multiple vehicles to complete their journeys, which is not realistic in the real situation. To ensure that passengers can take rides of only one driver to complete their trips, Di et al. [18] proposed a path-based nonlinear complementarity model with side constraints, where passengers take rides of ridesharing drivers of the same OD pair. Di et al. [18] also extended the ridesharing user equilibrium (RUE) model by considering the presence of high-occupancy toll (HOT) lane and found that the higher is the toll, the higher is the ridesharing rate and the lower is the total cost. Further, Di et al. [19] reformulated Xu et al.’s [17] model into link-node based RUE model and studied the deployment of high-occupancy toll (HOT) lanes. The results show that building HOT lanes may be effective to facilitate ridesharing. Considering the passengers’ OD pairs may differ from the ridesharing drivers’ OD pairs in reality, Li et al. [20] formulated a restricted path-based nonlinear complementarity model and explored whether Braess paradox could be avoided when HOT lanes are built in the context of ridesharing. The numerical examples show that Braess paradox disappeared under some conditions. Besides, Ma et al. [11] introduced an OD-based surge pricing strategy into a RUE model and found that the pricing strategy could eliminate the drivers’ behavior of deliberate detours. Li and Liu [12] built a RUE model which considers the travelers’ matching decisions. Sufficient conditions for matching failure were derived and a heuristic routing algorithm was proposed to run the model on large networks. Bahat et al. [36] incorporated the mode choice model into the traffic assignment model to quantify the ridesharing market share and found that the number of ridesharing drivers is the key to the growth of the ridesharing market. To explore how the total route cost and time are affected by the use of HOV lanes and toll savings, Wang et al. [37] modified the optimal route problem for ridesharing to a pickup and delivery problem considering the change in passenger travel time and toll cost due to vehicle load. All of the above studies assume that travelers are homogeneous with their VOTs, and none of them explicitly examine the impact of travelers’ VOTs on their ridesharing behaviors. It is well known that travelers are heterogeneous with their VOTs due to their socioeconomic characteristics and trip purpose [13,14], and travelers’ VOTs may vary over time. Ignoring the heterogeneity of VOTs has been confirmed to mislead evaluation of congestion pricing [38,39,40]. Therefore, travelers with heterogeneous VOTs may exhibit behavioral differences in paths and modes choices and ignoring the heterogeneity of VOTs in ridesharing may misestimate the spatial distribution of traffic congestion.

## 3. Model Formulation

### 3.1. Assumptions

To facilitate the presentation of the model formulating, several assumptions should be made in this paper as follows.

- Travelers/users are heterogeneous in terms of their VOTs and can be categorized into finite number of classes according to their VOTs.
- Each traveler owns a vehicle, and they each choose one of the three travel modes to complete his/her trips, i.e., solo driver(s) (SDs), ridesharing driver(s) (RDs), and ridesharing passenger(s) (RPs).
- Each RP takes a ride from only one RD, however each RD may pick up more than one RPs from the same OD pair. The RP’s OD pair may be different from the matched RD’s OD pair.
- The vehicles are uniform, and the vehicle capacity is limited and predetermined.

### 3.2. Model Description

**Figure 1:**
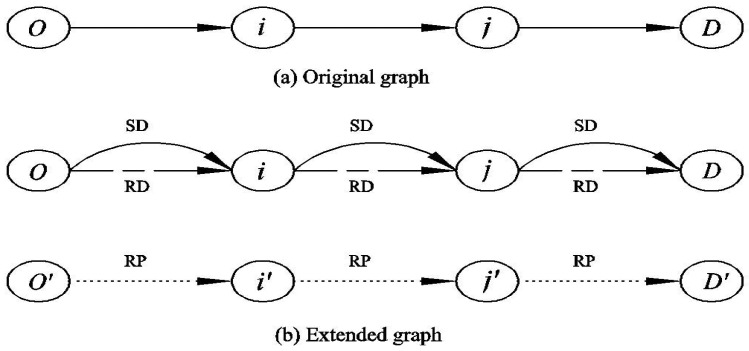
Graph extension.

Modeling traffic assignment with ridesharing should separate passengers’ flows from vehicular flows, because travelers who choose to be passengers have no contributions to congestion but can induce vehicular flow [41]. To distinguish flows of different travel modes in a same link, Xu et al. [17] extended the network (Figure 1), where the nodes were split into two and the links were split into three to separate solo drivers (SD), ridesharing drivers (RD) and ridesharing passengers (RP). Xu’s model assumed that SDs and RDs can interchange their roles at each node while passengers must remain their roles throughout their trips, which implies that travelers who choose to be passengers may have to transfer several vehicles to complete their trips. Therefore, the RPs’ network is disconnected, as shown in Figure 1. From the modeling perspective, the network is unchanged, while the corresponding variables are copied. Considering that passengers may be reluctant to ride in another vehicle in reality, Di et al. [18] and Li et al. [20] used distinguishing path-flow variables to separate these modes flows. Meanwhile, Li et al.’s [20] model takes into account the fact that RDs’ OD pair may be different from those of the matched RPs. This paper uses path-flow variables similar to those of Li et al. [20] to model ridesharing user equilibrium with heterogenous VOTs.

**Figure 2:**
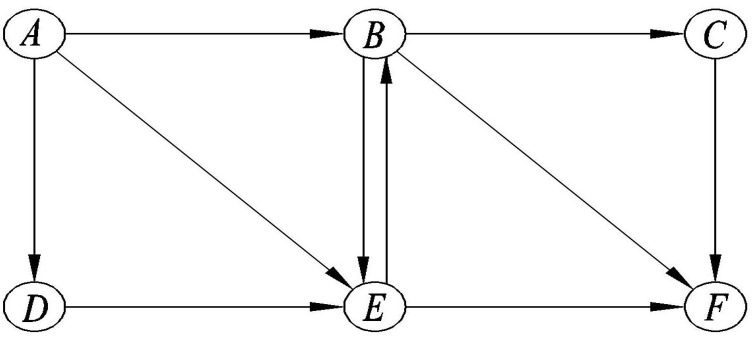
Paths of ridesharing matching graph.

Given a directed transportation network G=(N,A), *N* denotes the set of nodes and *A* is the set of directed links/arcs of the network. Let *W* denote the set of OD pairs and $R_{w}$ the set of all paths between the OD pair w∈W. Then, the set of all paths for all OD pairs can be defined as $R=\cup_{w\in W}R_{w}$. Travelers between each OD pair w∈W can be divided into a finite number of classes according to their VOTs. Let *M* denote the number of travelers classes and *m* a typical traveler class, m=1,2,…,M. Let $f_{r}^{m,\mathrm{SD}}$ denote the number of SDs in class *m* on path *r*, $f_{\mathrm{sr}}^{m,\mathrm{RD}}$ the number of RDs in class *m* on path *r* who travel with RPs on its sub-path *s*, and $f_{\mathrm{rp}}^{m,\mathrm{RP}}$ the number of RPs in class *m* on path *r* who share ride with RDs on its parent-path *p*, where $r\in R_{w}$, $s\in R_{l}$, $p\in R_{k}$, w∈W, l∈W, k∈W, ${▵}_{sr}=1$, ${▵}_{rp}=1$, and m=1,2,…,M. Path p∈R is a sub-path of path q∈R means the set of links of path *p* is contained in the set of links of path *q*. Correspondingly, the path *q* is the parent-path of path *p*. To express those paths relations in mathematical, ▵ denotes a matrix of size |R|×|R| whose element ${▵}_{pq}$ equals 1 if path *p* is a sub-path of path *q* and it is zero otherwise. Particularly, ${▵}_{pp}=1$ denotes that path *p* is the sub-path of itself. The path variables defined above actually indicate that the OD pair of RPs must be included in the path of the matched RDs, otherwise an RP could not complete his or her journey. For clarity, the feasible paths are further illustrated using the example in Figure 2. Consider the network in Figure 2 with two OD pair, $w_{1}=\left(A,F\right)$ and $w_{2}=\left(B,F\right)$. Passengers between $w_{2}$ have three options of routes, (B,F), (B,C,F), and (B,E,F). Note that, except for taking rides of RDs between $w_{2}$, these passengers can also carpool with RDs between $w_{1}$ who are traveling on routes (A,B,C,F), (A,B,F),(A,B,E,F), (A,E,B,F), and (A,E,B,C,F). Travelers between $w_{1}$ who choose to travel on routes (A,D,E,F) and (A,E,F) cannot share rides with travelers between $w_{2}$, since their travel routes do not include the RPs’ origin points between $w_{2}$.

Let $v_{a}^{m,\mathrm{SD}}$, $v_{a}^{m,\mathrm{RD}}$ be the flows of SDs and RDs of class *m* on link *a*, which can be calculated as follows,

(1a) $$\begin{array}{cc} \; & v_{a}^{m,\mathrm{SD}}=\sum_{w\in W}\sum_{r\in R_{w}}\delta_{ar}f_{r}^{m,\mathrm{SD}},\forall a\in A,m=1,2,\cdots ,M \end{array}$$

(1b) $$\begin{array}{cc} v_{a}^{m,\mathrm{RD}}= & \sum_{w_{W}}\sum_{r\in R_{w}}\sum_{s\in \{t\in R_{l},l\in W,{▵}_{tr}=1\}}\delta_{ar}f_{sr}^{m,\mathrm{RD}},\forall w\in W,m=1,2,\cdot \cdot \cdot ,M \end{array}$$

where $\delta_{ar}$ equals 1 if link *a* is on path *r* and it is zero otherwise.

Furthermore, RPs have no direct impact on road congestion, thus the vehicular flow on link *a* can be calculated as

(2) $$v_{a}=\sum_{m=1}^{M}\left(v_{a}^{m,\mathrm{SD}}+v_{a}^{m,\mathrm{RD}}\right),\forall a\in A$$

Given the demands {$D_{w}^{m},w\in W,m=1,2,\cdots ,M$}, the flow conservation becomes as follows,

(3) $$\sum_{r\in R_{w}}\left\{f_{r}^{m,\mathrm{SD}}+\sum_{s\in \{t\in R_{l},l\in W,{▵}_{tr}=1\}}f_{sr}^{m,\mathrm{RD}}+\right.\left.\sum_{p\in \{q\in R_{l},l\in W,{▵}_{pq}=1\}}f_{rp}^{m,\mathrm{RP}}\right\}=D_{w}^{m}$$

The first item in the bracket of Equation (3) represents all SDs in class *m* on path *r*. The second item represents all RDs in class *m* on path *r* who travel with RPs on its all sub-path (*s* is sub-path of *r*). The third item represents all RPs in class *m* on path *r* who share rides with RDs on its entire parent-path (*p* is the parent-path of *r*). Equation (3) actually implies that the demand for each travel mode in each class is endogenously determined by the modes.

In the conventional traffic assignment models, one unit of travelers is served by only one unit of vehicular flow, while, in the context of ridesharing, one unit of vehicular flow may serve for several units of travelers. Xu et al. [17] added link-based variable constraints into traffic assignment model to reflect the ride-matching relationships between drivers and riders at UE. Similar path-based variable constraints were added to formulate path-based RUE models [11,12,18,19,20]. This paper attempts to explore how the travelers’ VOTs affect their ridesharing behaviors. The VOT of a specific traveler may be difficult to identify in reality, that is, travelers with different VOTs might share the same vehicle to complete their trips. Therefore, the ride-matching constraints are modified as follows,

(4a) $$\begin{array}{cc} \; & \sum_{m=1}^{M}f_{sr}^{m,\mathrm{RD}}\leq \sum_{m=1}^{M}f_{sr}^{m,\mathrm{RP}},\forall r\in R_{w},s\in R_{l},w\in W,l\in W,{▵}_{sr}=1 \end{array}$$

(4b) $$\begin{array}{cc} \; & \sum_{m=1}^{M}f_{sr}^{m,\mathrm{RP}}\leq \varpi \sum_{m=1}^{M}f_{sr}^{m,\mathrm{RD}},\forall r\in R_{w},s\in R_{l},w\in W,l\in W,{▵}_{sr}=1 \end{array}$$

where ϖ denotes the vehicle capacity. Inequality (4a) ensures that the number of RPs of all classes on the sub-path is enough to satisfy demands of RDs of all classes on the parent-path. Inequality (4b) ensures that the number of rides provided by RDs of all classes on the parent-path is enough to satisfy demands of RPs of all classes on the sub-path.

### 3.3. Cost Functions

Unlike user equilibrium (UE), by which all travelers experience the same type of travel costs, travelers who choose different travel modes experience different types of travel costs in ridesharing user equilibrium (RUE). The travel cost functions of each type are defined as below.

#### 3.3.1. Congestion Cost (Travel Time)

All travelers experience congestion cost. The following BPR (Bureau of Public Roads) function [11,42,43] is used to calculate the link congestion cost as follow

(5a) $$\begin{array}{c} t_{a}\left(v_{a}\right)=t_{a}^{0}\left(1+\alpha {\left(\frac{v_{a}}{c_{a}}\right)}^{l}\right),a\in A \end{array}$$

where $t_{a}^{0}$ denotes the free-flow time on link a∈A, $c_{a}$ is the link capacity, and α and *l* are parameters related to congestion.

#### 3.3.2. Driving Cost

Travelers who choose to be drivers inevitably experience fuel cost, depreciation cost, and, possibly, toll. These costs can be unified as driving cost. The driving cost has been taken into account by several RUE models [11,36], and it is assumed to be a constant. Intuitively, more drivers on the link will slow down the vehicular speed, thus this may result in more fuel consumption, vehicle depreciation, or, possibly, toll. Therefore, the driving cost can be defined as follows,

(5b) $$\begin{array}{c} \varphi_{a}\left(v_{a}\right)=\theta {\left(\frac{v_{a}}{c_{a}}\right)}^{n},a\in A \end{array}$$

where $\varphi_{a}\left(v_{a}\right)$ is the driving cost function on link a∈A. θ denotes the price of fuel per unit. *n* is parameter related to congestion.

#### 3.3.3. Inconvenience Cost

One of the major obstacles to join in ridesharing is that the ridesharing participants have to experience inconvenience cost. The inconvenience cost includes time of matching response by the platform, time for picking up/dropping off (getting on/getting off), and even time for waiting for the desired RPs (RDs) to appear. The inconvenience costs of RDs and RPs are in the same form as those in [17,20], which are defined, respectively, as follows

(5c) $$\begin{array}{c} \begin{array}{cc} I_{sr}^{\mathrm{RD}}(f_{sr}^{1,\mathrm{RD}},\cdots ,f_{sr}^{M,\mathrm{RD}}, & f_{sr}^{1,\mathrm{RP}},\cdots ,f_{sr}^{M,\mathrm{RP}})=\gamma^{\mathrm{RD}}\left(\sum_{m=1}^{M}f_{sr}^{m,\mathrm{RP}}+\sum_{m=1}^{M}f_{sr}^{m,\mathrm{RD}}\right)N_{sr}^{\mathrm{RP}},\\ \; & \forall r\in R_{w},s\in R_{l},w\in W,l\in W,{▵}_{sr}=1 \end{array} \end{array}$$

(5d) $$\begin{array}{c} \begin{array}{cc} I_{rp}^{\mathrm{RP}}(f_{rp}^{1,\mathrm{RD}},\cdots ,f_{rp}^{M,\mathrm{RD}}, & f_{rp}^{1,\mathrm{RP}},\cdots ,f_{rp}^{M,\mathrm{RP}})=\gamma^{\mathrm{RP}}\left(\sum_{m=1}^{M}f_{rp}^{m,\mathrm{RP}}+\sum_{m=1}^{M}f_{rp}^{m,\mathrm{RD}}\right)N_{rp}^{\mathrm{RP}}\\ \; & \forall r\in R_{w},p\in R_{l},w\in W,l\in W,{▵}_{rp}=1 \end{array} \end{array}$$

Equations (5c) and (5d) mean that more ridesharing participants will result in more inconvenience cost, where $\gamma^{\mathrm{RD}}$ and $\gamma^{\mathrm{RD}}$ are positive parameters that reflect the intolerance to inconvenience for RDs and RPs. $N_{sr}^{\mathrm{RP}}$ is the number of links traveling with RPs.

#### 3.3.4. Compensations of RDs and Ridesharing Fees of RPs

Compensations of RDs are fees of RPs which pay to RDs by the transportation network company. The compensations of RDs and the ridesharing fees of RPs are in the same form as those in [20]. Compensation is a key point that benefits solo drivers to participate in ridesharing activities. Intuitively, the more RPs are in a vehicle, the more compensation there is for a RD; conversely, the more RDs are on the path, the fewer RPs there are in each vehicle, thus the lower is compensation for each RD. On the other hand, the more RPs there are in a vehicle, the lower are the fees for these RPs, and the more RDs there are on the path, the fewer RPs there are in each vehicle, thus the greater are the fees experienced by each RP. Therefore, the compensation function of RDs and the ridesharing fee function of RPs are defined, respectively, as follows

(5e) $$\begin{array}{c} \begin{array}{cc} R_{sr}^{\mathrm{RD}}(f_{sr}^{1,\mathrm{RD}},\cdots ,f_{sr}^{M,\mathrm{RD}}, & f_{sr}^{1,\mathrm{RP}},\cdots ,f_{sr}^{M,\mathrm{RP}})=\chi \left(\omega^{\mathrm{RD}}\sum_{m=1}^{M}f_{sr}^{m,\mathrm{RP}}-\sum_{m=1}^{M}f_{sr}^{m,\mathrm{RD}}\right)N_{sr}^{\mathrm{RP}}\\ \; & \forall r\in R_{w},s\in R_{l},w\in W,l\in W,{▵}_{sr}=1 \end{array} \end{array}$$

(5f) $$\begin{array}{c} \begin{array}{cc} R_{rp}^{\mathrm{RP}}(f_{rp}^{1,\mathrm{RD}},\cdots ,f_{rp}^{M,\mathrm{RD}}, & f_{rp}^{1,\mathrm{RP}},\cdots ,f_{rp}^{M,\mathrm{RP}})=\chi \left(\sum_{m=1}^{M}f_{rp}^{m,\mathrm{RD}}-\omega^{\mathrm{RP}}\sum_{m=1}^{M}f_{rp}^{m,\mathrm{RP}}\right)N_{rp}^{\mathrm{RP}}\\ \; & \forall r\in R_{w},p\in R_{l},w\in W,l\in W,{▵}_{rp}=1 \end{array} \end{array}$$

where χ is the guiding price for taking/sharing rides and $\omega^{\mathrm{RD}}$ and $\omega^{\mathrm{RP}}$ are positive parameters used to control the operating cost of the transportation network company.

#### 3.3.5. Path Cost Function

In the context of ridesharing, SDs experience congestion cost and driving cost. RDs experience congestion cost, driving cost, inconvenience cost, and compensation. RPs experience congestion cost, inconvenience cost, and ridesharing fees. These costs can be grouped into two categories: time cost and monetary cost. The time cost for SDs refers to congestion time. For RDs and RPs, the time cost refers to congestion time and inconvenience cost. Monetary cost for SDs is driving cost. For RDs, monetary cost consists of driving cost and compensation. For RPs, monetary cost refers to ridesharing fees. The time cost and the monetary cost could be converted into time-based or monetary-based cost by introducing a parameter of VOT according to Yang and Huang [44] and Huang and Li [45].

Let $\beta =\{\beta^{m},m=1,2,\cdots ,M\}$ be the set of corresponding VOTs for all traveler classes, where $\beta^{m}$ is the average VOT for travelers of class *m*. Let $C_{r}^{m,\mathrm{SD}}$, $C_{sr}^{m,\mathrm{RD}}$, and $C_{rp}^{m,\mathrm{RP}}$ be the cost function of SDs, RDs, and RPs in class *m* on path *r*, respectively, as follows.

Travel cost of SDs in class *m* on path *r*,

(6a) $$\begin{array}{c} \begin{array}{c} C_{r}^{m,\mathrm{SD}}=\sum_{a\in A}t_{a}\left(v_{a}\right)\delta_{ar}+\frac{1}{\beta^{m}}\sum_{a\in A}\varphi_{a}\left(v_{a}\right)\delta_{ar}\forall r\in R_{w},w\in W,m=1,2,\cdots ,M \end{array} \end{array}$$

Travel cost of RDs in class *m* on path *r*,

(6b) $$\begin{array}{c} \begin{array}{cc} \; & C_{sr}^{m,\mathrm{RD}}=\sum_{a\in A}t_{a}\left(v_{a}\right)\delta_{ar}+I_{sr}^{\mathrm{RD}}+\frac{1}{\beta^{m}}\left(\sum_{a\in A}\varphi_{a}\left(v_{a}\right)\delta_{ar}-R_{sr}^{\mathrm{RD}}\right)\\ \; & \forall r\in R_{w},s\in R_{l},w\in W,l\in W,\Delta_{sr}=1,m=1,2,\cdots ,M \end{array} \end{array}$$

Travel cost of RPs in class *m* on path *r*,

(6c) $$\begin{array}{c} \begin{array}{cc} \; & C_{rp}^{m,\mathrm{RP}}=\rho^{m}\sum_{a\in A}t_{a}\left(v_{a}\right)\delta_{ar}+I_{rp}^{\mathrm{RP}}+\frac{1}{\beta^{m}}R_{rp}^{\mathrm{RP}}\\ \forall r\in R_{w}, & p\in R_{l},w\in W,l\in W,{▵}_{rp}=1,m=1,2,\cdots ,M \end{array} \end{array}$$

where $\rho^{m}$ is the measure parameter of intolerance to congestion time for RPs in class *m*. $I_{sr}^{\mathrm{RD}}$ and $I_{sr}^{\mathrm{RP}}$ are simplified representations of Equations (5c) and (5d), respectively. $R_{sr}^{\mathrm{RD}}$ and $R_{sr}^{\mathrm{RD}}$ are simplified representations of Equations (5e) and (5f), respectively. We assume $\rho^{m}<1$, which means RPs experience less congestion time than drivers, since RPs can do their business and need not pay attention to the traffic. Moreover, travelers with high VOTs usually have higher requirements for the reliability of travel time [46], thus their aversion to congestion time may be greater than that of lower VOTs when give up driving.

The existence of side Constraints (4a) and (4b) make RUE fall into the category of UE with side constraints [18]. According to Larsson and Patriksson [47], UE with side constraint paths also incur costs due to the side constraints. Let $\eta_{sr}^{+}$ and $\eta_{sr}^{-}$ be multipliers of Constraints (4a) and (4b), respectively. Then, the generalized costs of travelers in each classes can be written as follows:

(7a) $$\begin{array}{c} {\tilde{C}}_{r}^{m,\mathrm{SD}}=\sum_{a\in A}\delta_{ar}t_{a}\left(v_{a}\right)+\frac{1}{\beta^{m}}\sum_{a\in A}\delta_{ar}\varphi_{a}\left(v_{a}\right),\forall r\in R_{w},w\in W,m=1,2,\cdots ,M \end{array}$$

(7b) $$\begin{array}{c} \begin{array}{cc} {\tilde{C}}_{sr}^{m,\mathrm{RD}}= & \sum_{a\in A}\delta_{ar}t_{a}\left(v_{a}\right)+I_{sr}^{\mathrm{RD}}+\frac{1}{\beta^{m}}\left(\sum_{a\in A}\delta_{ar}\varphi_{a}\left(v_{a}\right)-R_{sr}^{\mathrm{RD}}+\left(\eta_{sr}^{+}-\varpi \eta_{sr}^{-}\right)\right)\\ \; & \forall r\in R_{w},s\in R_{l},w\in W,l\in W,\Delta_{sr}=1,m=1,2,\cdots ,M \end{array} \end{array}$$

(7c) $$\begin{array}{c} \begin{array}{cc} \; & {\tilde{C}}_{sr}^{m,\mathrm{RP}}=\rho^{m}\sum_{a\in A}\delta_{ar}t_{a}\left(v_{a}\right)+I_{sr}^{\mathrm{RP}}+\frac{1}{\beta^{m}}\left(R_{sr}^{\mathrm{RP}}-\left(\eta_{sr}^{+}-\eta_{sr}^{-}\right)\right)\\ \; & \forall r\in R_{w},s\in R_{l},w\in W,l\in W,{▵}_{sr}=1,m=1,2,\cdots ,M \end{array} \end{array}$$

where, $\eta_{sr}^{+}$ and $\eta_{sr}^{-}$ are positive only when Inequalities (4a) and (4b) are binding, respectively. $\eta_{sr}^{+}$ and $\eta_{sr}^{-}$ can be considered a price adjustment at RUE [11]. Constraints (4a) and (4b) describe the actual supply and demand of ridesharing participants, while the potential supply and demand are suppressed by $\eta_{sr}^{+}$ and $\eta_{sr}^{-}$. Namely, when there are more potential RDs, $\eta_{sr}^{+}$ will be positive, which means a discount in the price for RPs extracting from RDs. Conversely, when there are more potential RPs, $\eta_{sr}^{-}$ will be positive, which denotes charging a premium price charging from RPs to subsidize RDs.

### 3.4. Nonlinear Complementarity Formulation

At multiclass ridesharing user equilibrium (MRUE), no one in each class can improve his or her travel cost by unilaterally changing his or her mode or path. The MRUE can be formulated as a mixed nonlinear complementarity problem (NCP) as follows,

(8a) $$\begin{array}{c} 0\leq f_{r}^{m,\mathrm{SD}}⊥{\tilde{C}}_{r}^{m,\mathrm{SD}}-\pi_{w}^{m}\geq 0,\forall r\in R_{w},w\in W,m=1,2,\cdots ,M \end{array}$$

(8b) $$\begin{array}{c} \begin{array}{cc} \; & 0\leq f_{sr}^{m,\mathrm{RD}}⊥{\tilde{C}}_{sr}^{m,\mathrm{RD}}-\pi_{w}^{m}\geq 0,\\ \forall r\in R_{w},s\in R_{l}, & w\in W,l\in W,{▵}_{sr}=1,m=1,2,\cdots ,M \end{array} \end{array}$$

(8c) $$\begin{array}{c} \begin{array}{cc} \; & 0\leq f_{sr}^{m,\mathrm{RP}}⊥{\tilde{C}}_{sr}^{m,\mathrm{RP}}-\pi_{w}^{m}\geq 0,\\ \forall r\in R_{w},s\in R_{l}, & w\in W,l\in W,{▵}_{sr}=1,m=1,2,\cdots ,M \end{array} \end{array}$$

(8d) $$\begin{array}{c} \begin{array}{cc} \; & 0\leq \eta_{sr}^{+}⊥\left[\sum_{m=1}^{M}f_{sr}^{m,\mathrm{RP}}-\sum_{m=1}^{M}f_{sr}^{m,\mathrm{RD}}\right]\geq 0\\ \forall r\in R_{w}, & s\in R_{l},w\in W,l\in W,{▵}_{rp}=1,m=1,2,\cdots ,M \end{array} \end{array}$$

(8e) $$\begin{array}{c} \begin{array}{cc} \; & 0\leq \eta_{sr}^{-}⊥\left[\varpi \sum_{m=1}^{M}f_{sr}^{m,\mathrm{RD}}-\sum_{m=1}^{M}f_{sr}^{m,\mathrm{RP}}\right]\geq 0\\ \forall r\in R_{w}, & s\in R_{l},w\in W,l\in W,{▵}_{rp}=1,m=1,2,\cdots ,M \end{array} \end{array}$$

(8f) $$\begin{array}{c} \begin{array}{cc} \pi_{w}^{m}\mathrm{free}, & \sum_{r\in R_{w}}\left(f_{r}^{m,\mathrm{SD}}+\sum_{s\in \{t\in R_{l},l\in W,{▵}_{tr}=1\}}f_{sr}^{m,\mathrm{RD}}+\sum_{p\in \{q\in R_{l},l\in W,{▵}_{pq}=1\}}f_{rp}^{m,\mathrm{RP}}\right)-D_{w}^{m}=0\\ \; & \forall r\in R_{w},s\in R_{l},p\in R_{k},k\in W,w\in W,l\in W,m=1,2,\cdots ,M \end{array} \end{array}$$

where ⊥ denotes the inner product of two vectors is zero. Equations (8a)–(8c) represent the equilibrium constraints. Equations (8d) and (8e) are ride-matching constraints. Equation (8f) represents flow conservations.

## 4. The Equivalent Variational Inequality Formulation and Existence of the Model Solution

Let $f=\left[f_{r}^{1,\mathrm{SD}},\cdots ,f_{|R_{w}|}^{1,\mathrm{SD}},\cdots ,f_{|R_{w}|}^{M,\mathrm{SD}},f_{sr}^{1,\mathrm{RD}},\cdots ,f_{|R_{w}|}^{M,\mathrm{RD}},f_{rp}^{1,\mathrm{RP}},\cdots ,f_{|R_{w}|}^{M,\mathrm{RP}}\right]$ be the vector of path variables and Ω denotes the set of feasible paths, i.e.,

$$\Omega =\left\{f\geq 0\left|\begin{array}{c} \begin{array}{cc} & \sum_{r\in R_{w}}\left(f_{r}^{m,\mathrm{SD}}+\sum_{s\in \{t\in R_{l},l\in W,\Delta_{tr}=1\}}f_{sr}^{m,\mathrm{RD}}+\sum_{p\in \{q\in R_{l},l\in W,\Delta_{rq}=1\}}f_{rp}^{m,\mathrm{RP}}\right)-D_{w}^{m}=0\\ & \sum_{m=1}^{M}f_{sr}^{m,\mathrm{RP}}-\sum_{m=1}^{M}f_{sr}^{m,\mathrm{RD}}\geq 0\\ & \varpi \sum_{m=1}^{M}f_{sr}^{m,\mathrm{RD}}-\sum_{m=1}^{M}f_{sr}^{m,\mathrm{RP}}\geq 0\\ & \forall r\in R_{w},s\in R_{l},p\in R_{k},k\in W,w\in W,l\in W,{▵}_{sr}=1,m=1,2,\cdots ,M \end{array} \end{array}\right.\right.$$

Then, the equivalent variational inequality (VI) problem of the NCP (8) is as below.

Find a vector $f^{*}\in \Omega$ such that

(9) $$\begin{array}{c} \begin{array}{c} \sum_{w\in W}\sum_{r\in R_{w}}\sum_{m=1}^{M}C_{r}^{*,m,\mathrm{SD}}\left(f_{r}^{m,\mathrm{SD}}-f_{r}^{*m,\mathrm{SD}}\right)+\sum_{w\in W}\sum_{r\in R_{w}}\sum_{s\in \{t\in R_{l},l\in W,{▵}_{tr}=1\}}\sum_{m=1}^{M}C_{sr}^{*,m,\mathrm{RD}}(\\ f_{sr}^{m,\mathrm{RD}}-f_{sr}^{*m,\mathrm{RD}})+\sum_{w\in W}\sum_{r\in R_{w}}\sum_{p\in \{q\in R_{l},l\in W,{▵}_{pq}=1\}}\sum_{m=1}^{M}C_{rp}^{*,m,\mathrm{RP}}\left(f_{rp}^{m,\mathrm{RP}}-f_{rp}^{*m,\mathrm{RP}}\right)\geq 0 \end{array} \end{array}$$

**Theorem** **1.** *The solution of the VI (9) is equivalent to the solution of the NCP (8).*

**Proof of Theorem 1.**  Equation (9) is apparently equal to

(10) $$\begin{array}{c} \begin{array}{cc} \; & \sum_{w\in W}\sum_{r\in R_{w}}\sum_{m=1}^{M}C_{r}^{*,m,\mathrm{SD}}f_{r}^{m,\mathrm{SD}}+\sum_{w\in W}\sum_{r\in R_{w}}\sum_{s\in \{t\in R_{l},l\in W,{▵}_{tr}=1\}}\sum_{m=1}^{M}C_{sr}^{*,m,\mathrm{RD}}f_{sr}^{m,\mathrm{RD}}+\\ \; & \sum_{w\in W}\sum_{r\in R_{w}}\sum_{p\in \{q\in R_{l},l\in W,{▵}_{pq}=1\}}\sum_{m=1}^{M}C_{rp}^{*,m,\mathrm{RP}}f_{rp}^{m,\mathrm{RP}}\\ \geq \\ \; & \sum_{w\in W}\sum_{r\in R_{w}}\sum_{m=1}^{M}C_{r}^{*,m,\mathrm{SD}}f_{r}^{*m,\mathrm{SD}}+\sum_{w\in W}\sum_{r\in R_{w}}\sum_{s\in \{t\in R_{l},l\in W,{▵}_{tr}=1\}}\sum_{m=1}^{M}C_{sr}^{*,m,\mathrm{RD}}f_{sr}^{*m,\mathrm{RD}}+\\ \; & \sum_{w\in W}\sum_{r\in R_{w}}\sum_{p\in \{q\in R_{l},l\in W,{▵}_{pq}=1\}}\sum_{m=1}^{M}C_{rp}^{*,m,\mathrm{RP}}f_{rp}^{*m,\mathrm{RP}} \end{array} \end{array}$$

A vector $f^{*}$ is a solution to the VI if and only if $f^{*}$ is a solution of the following mathematical programming of the variable f,

(11) $$\begin{array}{c} \begin{array}{cc} \min_{f\in \Omega }\sum_{w\in W}\sum_{r\in R_{w}}\sum_{m=1}^{M} & C_{r}^{*,m,\mathrm{SD}}f_{r}^{m,\mathrm{SD}}+\sum_{w\in W}\sum_{r\in R_{w}}\sum_{s\in \{t\in R_{l},l\in W,{▵}_{tr}=1\}}\sum_{m=1}^{M}C_{sr}^{*,m,\mathrm{RD}}f_{sr}^{m,\mathrm{RD}}\\ \; & +\sum_{w\in W}\sum_{r\in R_{w}}\sum_{p\in \{q\in R_{l},l\in W,{▵}_{pq}=1\}}\sum_{m=1}^{M}C_{rp}^{*,m,\mathrm{RP}}f_{rp}^{m,\mathrm{RP}} \end{array} \end{array}$$

The Karush–Kuhn–Tucker (KKT) condition for the mathematical programming (11) is that

$$\left\{\begin{array}{c} 0\leq f_{r}^{*m,\mathrm{SD}}⊥{\tilde{C}}_{r}^{*m,\mathrm{SD}}-\pi_{w}^{m}\geq 0\\ 0\leq f_{sr}^{*m,\mathrm{RD}}⊥{\tilde{C}}_{sr}^{*m,\mathrm{RD}}-\pi_{w}^{m}\geq 0\\ 0\leq f_{sr}^{*m,\mathrm{RP}}⊥{\tilde{C}}_{sr}^{*m,\mathrm{RP}}-\pi_{w}^{m}\geq 0\\ 0\leq \eta_{sr}^{+}⊥\left[\sum_{m=1}^{M}f_{sr}^{*m,\mathrm{RP}}-\sum_{m=1}^{M}f_{sr}^{*m,\mathrm{RD}}\right]\geq 0\\ 0\leq \eta_{sr}^{-}⊥\left[\varpi \sum_{m=1}^{M}f_{sr}^{*m,\mathrm{RD}}-\sum_{m=1}^{M}f_{sr}^{*m,\mathrm{RP}}\right]\geq 0\\ \sum_{r\in R_{w}}\left(f_{r}^{*m,\mathrm{SD}}+\sum_{s\in \{t\in R_{l},l\in W,{▵}_{tr}=1\}}f_{sr}^{*m,\mathrm{RD}}+\sum_{p\in \{q\in R_{l},l\in W,{▵}_{pq}=1\}}f_{rp}^{*m,\mathrm{RP}}\right)-D_{w}^{m}=0 \end{array}\right.$$

which is exactly the NCP (8). □

**Theorem** **2.** *the VI (9) has at least one solution.*

**Proof of Theorem 2.**  Since the set Ω is a polyhedron and travel demand is fixed, the compactness and convexity of Ω are apparent. Moreover, the cost functions are all assumed to be continuous. According to Facchinei and Pang [48], there exists at least one solution. However, the uniqueness of the model solution cannot be guaranteed since we cannot expect the travel cost functions of each class to be strictly monotone. □

## 5. Numerical Experiments

**Figure 3:**
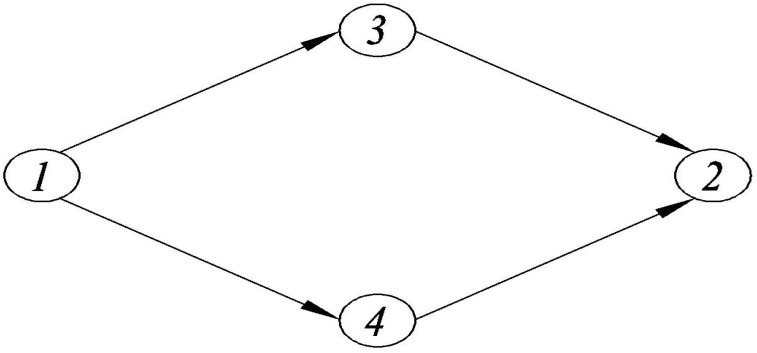
The test network with two OD pairs.

The topology of the test network is illustrated in Figure 3. In the test network, N={1,3,4,2}, A={(1−3),(3−2),(1−4),(4−2)}, and there are two OD pairs, from Node 1 to Node 2 ($w_{1}$) and from Node 3 to Node 2 ($w_{2}$). RDs between OD pair $w_{1}$ can pick up RPs from OD pair $w_{2}$.

Travelers between each OD pair are divided into *M* classes. It may be a hard job to investigate the VOTs of all travelers in a certain area. In general, the statistical distribution of their VOTs can be obtained through investigation and statistical analysis. To cover more possible situations, we assume that the distribution of VOTs follows lognormal distribution and is identical for all OD pairs [45,49]. Let f(τ) represent the continuous probability density function of VOTs as follows,

(12) $$\begin{array}{cc} f\left(\tau \right)=\frac{1}{\kappa \sqrt{2\pi }} & \tau^{-1}exp\left[-\frac{1}{2}{\left(\frac{ln\tau -\lambda }{\kappa }\right)}^{2}\right],0<\tau <\propto ,\kappa >0 \end{array}$$

where λ and κ are the mean and the standard deviation of lnτ, respectively.

Let $D_{w}$ be the demand of travelers between OD pair w∈W. $D_{w}$ is fixed and given. Let F(τ) be the cumulative distribution function of f(τ). All samples of VOTs are ranked from smallest to largest in terms of the value. Assume that $\tau_{\max}$ is the maximum sample of VOTs. Then, we can divide all travelers into *M* classes as follows,

(13) $$\left\{\begin{array}{c} \left[\tau^{m-1},\tau^{m}\right]=\left[\frac{m-1}{M}\tau_{\max},\frac{m}{M}\tau_{\max}\right],\forall m=1,2,\cdots ,M-1\\ \left[\tau^{m-1},\tau^{m}\right]=\left[\frac{m-1}{M}\tau_{\max},+\propto \right],m=M \end{array}\right.$$

(14) $$\left\{\begin{array}{c} D_{w}^{m}=D_{w}\left[F\left(\tau^{m}\right)-F\left(\tau^{m-1}\right)\right],\forall m=1,2,\cdots ,M-1\\ D_{w}^{m}=D_{w}\left[1-F(\tau^{m-1})\right],m=M \end{array}\right.$$

Obviously, the following is true,

(15) $$\sum_{m=1}^{M}D_{w}^{m}=D_{w},\forall w\in W$$

The average VOT $\beta_{m}\left(\beta_{m}>0\right)$ for travelers of class *m* can be computed by

(16) $$\left\{\begin{array}{cc} \beta_{m} & =\frac{\int_{\tau^{m-1}}^{\tau^{m}}\tau f\left(\tau \right)d\tau }{\int_{\tau^{m-1}}^{\tau^{m}}f\left(\tau \right)d\tau },\forall m=1,2,\cdots ,M-1\\ \beta^{m} & =\frac{\int_{\tau^{m-1}}^{+\propto }\tau f\left(\tau \right)d\tau }{\int_{\tau^{m-1}}^{+\propto }f\left(\tau \right)d\tau },m=M \end{array}\right.$$

The proposed MRUE (8) can be transformed into an optimization problem by the NCP-FB function, which is written as

(17) $$\phi \left(x,y\right):=\sqrt{x^{2}+y^{2}}-x-y$$

and the transformed optimization problem was calculated by applying the KNITRO 11.0 solver on MATLAB 2016a.

In this example, let M=2. Then, travelers between each OD pair are separated into two classes in term of their VOTs, which are distinguished as class high (Class *H*) and class low (Class *L*). Travelers in each class have three travel modes. Therefore, there are six roles of travelers when the traffic system reaches equilibrium, i.e., solo drivers in Class *L* (LSDs), ridesharing drivers in Class *L* (LRDs), ridesharing passengers in Class *L* (LRPs), solo drivers in Class *H* (HSDs), ridesharing drivers in Class *H* (HRDs), and ridesharing passengers in Class *H* (HRPs). All possible paths are listed in Table 1. Note that RPs on R21 and R22 can share rides with RDs on R19 and R20. *L* and *H* denote Class *L* and Class *H*, respectively.

**Remark** **1.** *It would be a challenging task to calculate the proposed RUE model on a large network. Firstly, the proposed model is path-based and non-additive, which requires path enumeration. Secondly, the numbers of sub-paths and the parent-paths increase explosively with the increase of the number of OD pairs and the topology of the network. Thirdly, modes separation and travelers’ classification also multiply the set of feasible paths. For example, consider a network with n OD pairs; assuming that each OD pair has k paths, the number of sub-paths and parent-paths between any two OD pairs is l, and travelers are divided into m classes, then the number of feasible paths is approximately equal to $3\times m\times (\left(n\times k\right)+\left(l\times C_{n}^{2}\right))$ and the number of multipliers are approximately equal to $\left(n\times k\right)+\left(l\times C_{n}^{2}\right)+m\times n$. Fortunately, some efficient path selection algorithms have been developed. The RUE model proposed by Ma et al. [11] was solved by using the column generation techniques and projection algorithm, and Li et al. [12] filtered out the paths which matched failure based on the ride-matching conditions proposed in the RUE model. However, using these methods to reduce the computational complexity of the RUE models requires that the model has certain properties, which is the further work of this paper.
*

### 5.1. Computational Result

Given $D_{w_{1}}=100$, $D_{w_{2}}=50$, $\tau_{\max}=10$, set $t_{a}^{0}=15$, $c_{a}=6$, α=0.04, l=2, θ=3, n=1, $\gamma^{\mathrm{RD}}=0.3$, $\gamma^{\mathrm{RP}}=0.3$, $\omega^{\mathrm{RD}}=0.6$, $\omega^{\mathrm{RP}}=0.4$, $\rho^{L}=0.8$, $\rho^{H}=0.85$, ϖ=2, χ=6, κ=0.1, and λ=1.6. Then, $\beta_{L}=4.6$, $\beta_{H}=5.4$ (*L* and *H* denote Class *L* and Class *H*, respectively) can be calculated by Equations (12) and (16). Table 2 lists the routes flows and the corresponding routes costs at UE.

Table 2 shows that, for each OD pair, the sum of the route flows of the three modes of each class is equal to the total demand of that class. Besides, for any OD pair, the cost of each used route of each class is equal to its minimum generalized cost of that class and the unused routes of each class is not less than the minimum generalized cost of that class. Note that 7.60 LRDs (R19) along with 0.67 HRDs (R20) between $w_{1}$ share rides with 12.24 LRPs (R21) and 4.29 HRPs (R22) between $w_{2}$. This illustrates that travelers with different VOTs and between different OD pairs do share rides with each other in this example. Another point to note is that the multipliers of matching Constraint (4b) are positive in this example, indicating that passengers need to pay extra fees for taking rides, since the potential demand of passengers is greater than the supply of ridesharing drivers under the parameter setting of this example. However, it does not mean that these multipliers are always positive. The multipliers of Constraint (4b) may be zero when the parameters are changed.

### 5.2. Sensitivity Analysis

**Figure 4:**
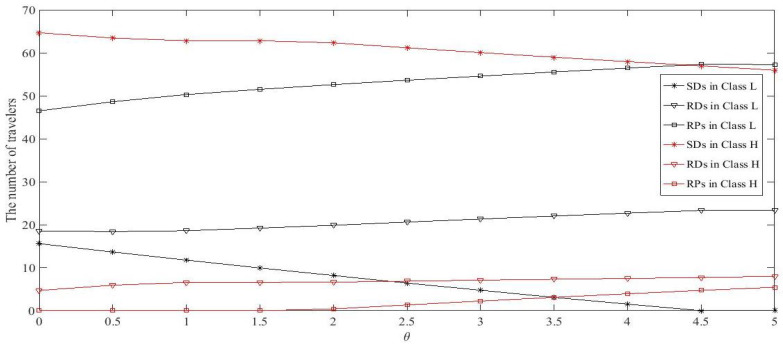
The change of mode choice for travelers in each class against θ.

Increasing θ means that drivers experience more driving cost. Intuitively, increasing the driving cost will result in the growth of passengers numbers because the travel cost of passengers decline relatively. Meanwhile, more passengers need more ridesharing drivers to share rides, thus the number of ridesharing drivers will also increase. Given $D_{w_{1}}=100$, $D_{w_{2}}=50$, and $\tau_{\max}=10$, the other parameters are set as follows: $t_{a}^{0}=15$, $c_{a}=6$, α=0.04, l=2, n=1, $\gamma^{\mathrm{RD}}=0.4$, $\gamma^{\mathrm{RP}}=0.4$, $\omega^{\mathrm{RD}}=0.6$, $\omega^{\mathrm{RP}}=0.4$, $\rho^{L}=0.8$, $\rho^{H}=0.85$, ϖ=2, χ=6, κ=0.1, and λ=1.6. Let θ range from 0 to 5. Figure 4 plots the number of travelers in each class choosing different modes with θ change at UE. Figure 4 shows that, as θ increases, the number of SDs decreases while RDs and RPs increases for both classes, as expected. Note that travelers in Class *L* are forced to give up driving alone when θ≥4.5, while the number of participants of ridesharing in Class *H* is increased slightly. This indicates that travelers with lower values of time are more sensitive to driving cost.

**Figure 5:**
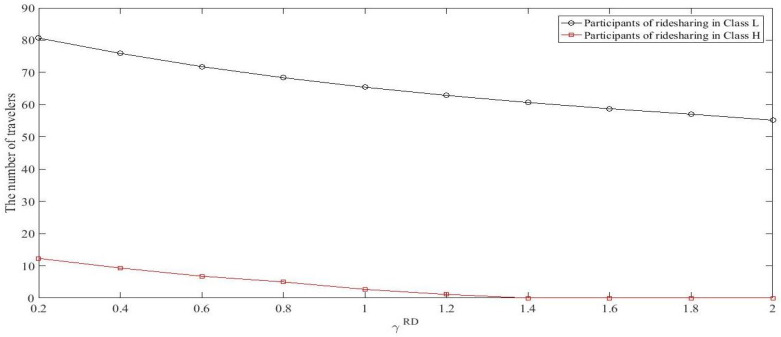
The number of ridesharing participants in each class against $\gamma^{\mathrm{RD}}$.

**Figure 6:**
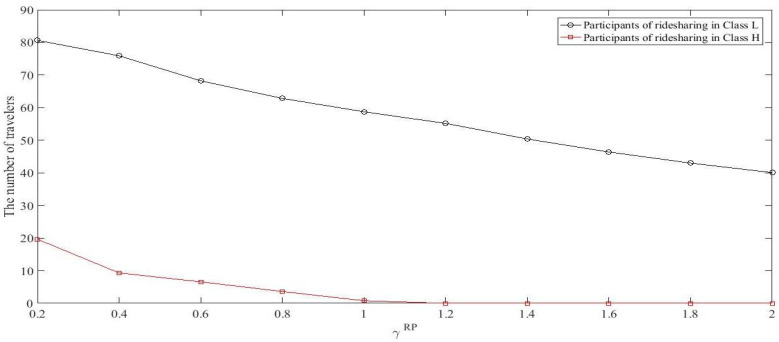
The number of ridesharing participants in each class against $\gamma^{\mathrm{RP}}$.

Increasing $\gamma^{\mathrm{RD}}$ and $\gamma^{\mathrm{RP}}$ means that the inconvenience cost grows. When the cost of ridesharing goes up for both ridesharing drivers and passengers, fewer travelers would like to participate in ridesharing. The main parameters are set as before, but varying some of them as follows. Given θ=3, let $\gamma^{\mathrm{RP}}=0.4$ while $\gamma^{\mathrm{RD}}$ ranges from 0.2 to 2, and let $\gamma^{\mathrm{RD}}=0.4$ while $\gamma^{\mathrm{RP}}$ ranges from 0.2 to 2. Figure 5 shows the number of ridesharing participants (the number of RDs plus RPs) in each class against $\gamma^{\mathrm{RD}}$ and Figure 6 shows the number of ridesharing participants in each class against $\gamma^{\mathrm{RP}}$ at RUE. As shown in Figure 5 and Figure 6, raising the inconvenience cost (whether it is the inconvenience cost of RDs or of RPs), the number of ridesharing participants decline for travelers in both classes. Besides, when the inconvenience coefficients increase to a certain value, it is almost a vacuum for travelers in Class *H* who participate in ridesharing activities. These indicate that travelers with higher values of time are more sensitive to inconvenience cost.

### 5.3. The Impact of VOTs on Ridesharing

**Figure 7:**
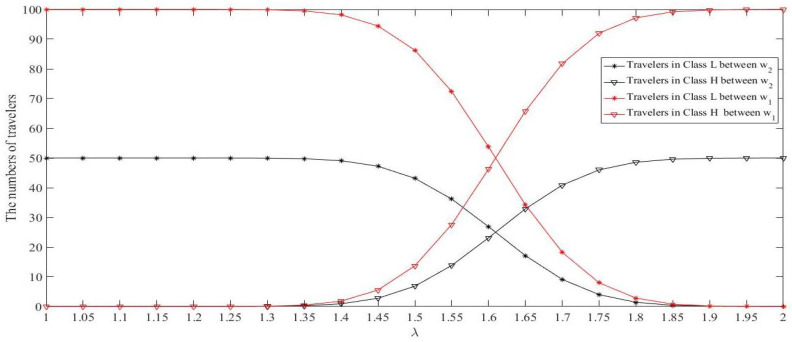
The number of travelers in each class between different OD pairs with λ changes.

**Figure 8:**
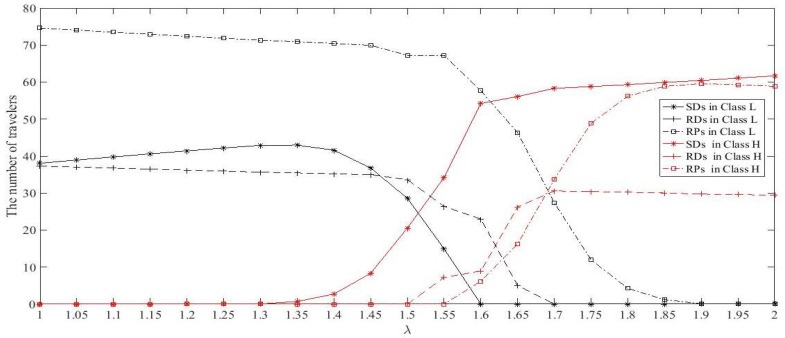
The number of total drivers and of participants of ridesharing with λ changes.

Given $D_{w_{1}}=100$, $D_{w_{2}}=50$, and $\tau_{\max}=10$, set $t_{a}^{0}=15$, $c_{a}=6$, α=0.04, l=2, θ=3, n=1, $\gamma^{\mathrm{RD}}=0.3$, $\gamma^{\mathrm{RP}}=0.3$, $\omega^{\mathrm{RD}}=0.6$, $\omega^{\mathrm{RP}}=0.4$, $\rho^{L}=0.8$, $\rho^{H}=0.85$, ϖ=2, and χ=6, κ=0.1. Let λ range from 1 to 2. According to Equations (12)–(16), the number of travelers in each classes between each OD pair against λ can be calculated, which is plotted in Figure 7. Figure 7 shows that, when λ<1.35 and λ>1.85, which is distinguished as Situation 1, there is only one class of travelers between each OD pair. In this situation, increasing λ means the travelers’ VOTs go up (Table 3) while the total demand of each OD pair is constant. When 1.35≤λ≤1.85, which is distinguished as Situation 2, there are two classes of travelers (Class *L* and Class *H*). In this situation, the number of travelers in Class *L* goes down while the number of travelers in Class *H* goes up as λ increases. Figure 8 plots the number of travelers in each class who choose different modes with λ change at RUE.

Figure 8 shows that, in Situation 1 (λ<1.35 and λ>1.85), the number of SDs increases while the number of RDs and RPs decreases as λ raises. This is because the RDs’ revenues are converted into less negative time cost as λ increases (i.e., the travelers’ VOTs increase). Thus, RDs’ generalized costs are larger than those of SDs. Therefore, some RDs and the matched RPs switch to SDs as λ increases. Note that, although the RPs’ generalized cost also decreases as λ increases, no RDs would switch to RPs. If so, it may result in more RDs to share rides, which contradicts the fact that some RDs switch to other modes. This indicates that travelers’ willingness of ridesharing might decline with their VOTs increase.

Figure 8 shows that, in Situation 2 (1.35≤λ≤1.85), when travelers in Class *L* dominates the total travel demand (1.35≤λ≤1.5), ridesharing occurs mainly among travelers in Class *L*, while travelers in Class *H* drive alone. However, when travelers in Class *H* dominate the total demand (1.7≤λ≤1.85), ridesharing occurs mainly among travelers in Class *H*, while travelers in Class *L* choose to be passengers. In the other case (1.55≤λ≤1.65), the number of ridesharing participants in Class *L* is larger than that in Class *H*.

The above results indicate that travelers with heterogeneous values of time show differences in their ridesharing behavior with the change of VOT distribution parameters.

### 5.4. The Impact of HOT Lane on Ridesharing

Building High-Occupancy Toll (HOT) lane is one of the most effective measures to encourage travelers to carpooling. Travelers who share rides usually use HOT lane freely. In general, to improve the utilization of HOT lane, solo drivers are also allowed to use HOT lanes by being charged a toll. This section explores how HOT lanes affect travelers’ ridesharing behaviors. The feasible path set has to be augmented to accommodate HOT lanes. Denote the set of all paths passing HOT lanes for OD pair w∈W as $R_{w}^{HOT}$. Toll for path *r*,$r\in R_{w}^{HOT}$ is calculated as

(18) $$z_{r}=\sum_{(i,j)\in G}\delta_{(i,j),r}z_{i,j}$$

where *G* denotes the set of links which are retrofitted to HOT lanes and $z_{i,j}$ denotes the toll on link (i,j). If a link (i,j) is retrofitted into general purpose (GP) lane and HOT lane, then the capacity of each type of lane is to be half as before (i.e., $c_{a}^{GP}=c_{a}^{HOT}=\frac{1}{2}c_{a}$). Since we assume HOT lanes only charge for solo drivers who use it, the generalized cost of solo drivers passing through HOT lanes can be calculated as

(19) $$\begin{array}{c} \begin{array}{cc} {\tilde{C}}_{r}^{m,\mathrm{SD}}=\sum_{a\in A} & \delta_{ar}t_{a}\left(v_{a}\right)+\frac{1}{\beta^{m}}\left(\sum_{a\in A}\delta_{ar}\varphi_{a}\left(v_{a}\right)+z_{r}\right)\\ \forall r\in R_{w}^{HOT}, & w\in W,m=1,2,\cdots ,M \end{array} \end{array}$$

**Figure 9:**
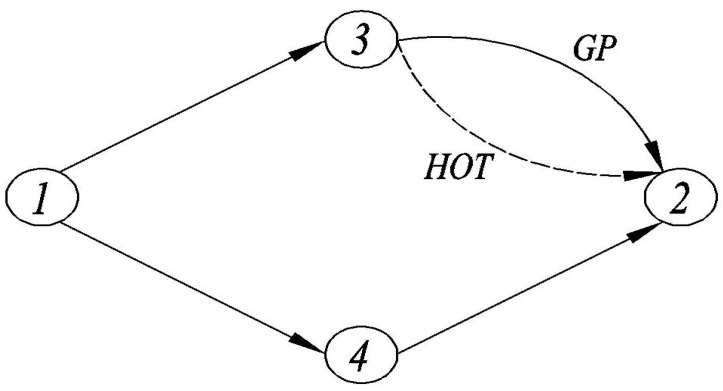
The test network with HOT lane.

**Figure 10:**
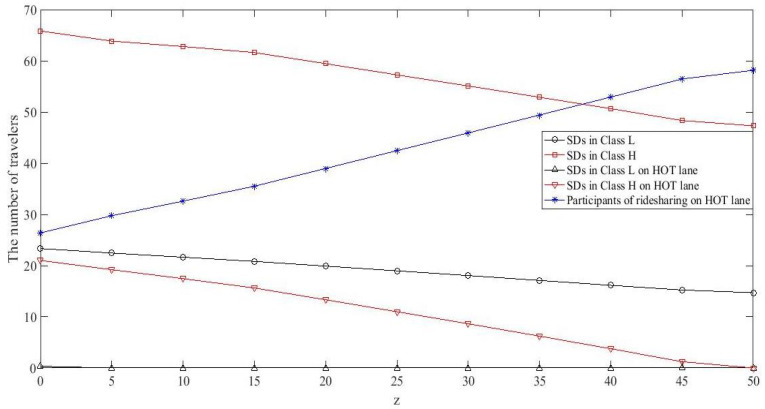
Several statistical results against $z_{a}$ at UE.

Figure 9 presents the test network in which link 3-2 is retrofitted into a GP lane and a HOT lane. Table 4 shows the path set of all the feasible path. Note that RPs on R21 and R22 can share rides with RDs on R19 and R20, and RDs on R37 and R38 can travel with RPs on R37 and R38. Given $D_{w_{1}}=100$, $D_{w_{2}}=50$, and $\tau_{\max}=10$, let $c_{a}=8$, χ=5, $t_{a}^{0}=10$, α=0.04, l=2, n=1, $\gamma^{\mathrm{RD}}=0.5$, $\gamma^{\mathrm{RP}}=0.5$, $\omega^{\mathrm{RD}}=0.6$, $\omega^{\mathrm{RP}}=0.4$, $\rho^{L}=0.8$, $\rho^{H}=0.85$, ϖ=2, θ=1, κ=0.1, and λ=1.6. By varying the toll *z* of HOT lane from 0 to 50, several statistical results at UE are plotted in Figure 10. Figure 10 shows that the number of total SDs in each class goes down while the number of ridesharing participants goes up on HOT lane, as the toll increases. More explicitly, the number of HSDs, especially those using the HOT lane, goes down dramatically. This is because the HSDs on the HOT lane shift to the GP lane due to the toll increasing on the HOT lane. Meanwhile, the LSDs on the GP lane switch to LRDs or LRPs and shift to travel on HOT lane, since the congestion of the GP lane cost goes up and HOT lane charges freely for ridesharing participants. This result is consistent with the impact of driving cost on travelers’ ridesharing behaviors in Section 5.2, since an increase in road pricing implies an increase in driving cost. In summary, building HOT lanes can effectively attract travelers to ridesharing.

## 6. Conclusions

This paper explores the relation between travelers’ values of time and their ridesharing behaviors on the congested network. A path-based ridesharing traffic assignment model is proposed by considering travelers with heterogeneous values of time. A more realistic ride-matching constraint was added in the proposed model, which captures the fact that travelers with different values of time can share trips together. In addition, the model solution was proved to be exist. The impact of high-occupancy toll lanes on ridesharing was further explored by amplifying the path set with high-occupancy toll lanes. Numerical examples were conducted on a simple network. The calculation results show that the model solution satisfies the Wardrop UE principle and the proposed model captures the main ridesharing characteristics of travelers. Besides, travelers with lower values of time are sensitive to the driving cost, while those with higher values of time are sensitive to the inconvenience cost. Furthermore, travelers with different values of time show differences in their ridesharing behavior with the change of value of time distribution parameters, which indicates the single-class ridesharing traffic assignment model may miscalculate the ridesharing scale of users. Finally, high-occupancy toll lanes can effectively attract travelers to participate in ridesharing.

Future works may be extended in several ways. Firstly, from the view of modeling, it is necessary to develop an effective solution algorithm based on non-additive path cost. At the same time, it is equally important to consider the impact of the nonlinear relationship between time cost and monetary cost on ridesharing activities. These improvements will help design reasonable network mechanisms to promote ridesharing. Secondly, we analyzed the rationality of the cost functions, such as driving cost, inconvenience cost, compensation/ridesharing fees, etc., and these functions are in line with our intuition. However, the more accurate forms of these cost functions still need to be found through developing effective machine learning methods and using the massive traffic data, as with the BPR formula. Finally, the assumption of rational travelers may be relaxed, since many empirical studies have shown that travelers’ travel decisions are influenced by psychological factors, e.g., regret, loss aversion, and limited cognition. It is still unclear how these psychological activities affect travelers’ ridesharing behaviors. Besides, how users perceive the social dimensions of sharing time and space with strangers is still unclear, too.

## Figures and Tables

**Table 1:** The enumerated paths of the test network when *M* = 2.

OD Pair $w_{1}$					OD Pair $w_{2}$	
Path 1-3-2		Path 1-4-2			Path 3-2	
R1	(1-3${}^{L,\mathrm{SD}}$,3-2${}^{L,\mathrm{SD}}$)	R7	(1-4${}^{L,\mathrm{SD}}$,4-2${}^{L,\mathrm{SD}}$)		R13	(3-2${}^{L,\mathrm{SD}}$)
R2	(1-3${}^{H,\mathrm{SD}}$,3-2${}^{H,\mathrm{SD}}$)	R8	(1-4${}^{H,\mathrm{SD}}$,4-2${}^{H,\mathrm{SD}}$)		R14	(3-2${}^{H,\mathrm{SD}}$)
R3	(1-3${}^{L,\mathrm{RD}}$,3-2${}^{L,\mathrm{RD}}$)	R9	(1-4${}^{L,\mathrm{RD}}$,4-2${}^{L,\mathrm{RD}}$)		R15	(3-2${}^{L,\mathrm{RD}}$)
R4	(1-3${}^{H,\mathrm{RD}}$,3-2${}^{H,\mathrm{RD}}$)	R10	(1-4${}^{H,\mathrm{RD}}$,4-2${}^{H,\mathrm{RD}}$)		R16	(3-2${}^{H,\mathrm{RD}}$)
R5	(1-3${}^{L,\mathrm{RP}}$,3-2${}^{L,\mathrm{RP}}$)	R11	(1-4${}^{L,\mathrm{RP}}$,4-2${}^{L,\mathrm{RP}}$)		R17	(3-2${}^{L,\mathrm{RP}}$)
R6	(1-3${}^{H,\mathrm{RP}}$,3-2${}^{H,\mathrm{RP}}$)	R12	(1-4${}^{H,\mathrm{RP}}$,4-2${}^{H,\mathrm{RP}}$)		R18	(3-2${}^{H,\mathrm{RP}}$)
R19	(1-3${}^{L,\mathrm{SD}}$, 3-2${}^{L,\mathrm{RD}}$)				R21	(3-2${}^{L,\mathrm{RP}\;*}$)
R20	(1-3${}^{H,\mathrm{SD}}$, 3-2${}^{H,\mathrm{RD}}$)				R22	(3-2${}^{H,\mathrm{RP}\;*}$)

* Note that RPs on R21 and R22 can share rides with RDs on R19 and R20. L and H denote Class L and Class H, respectively.

**Table 2:** The route flows and corresponding generalized route cost at RUE.

OD Pair 1						OD Pair 2		
Path 1-3-2			Path 1-4-2			Path 3-2		
Extended Route	Route Flow	Generalized Cost	Extended Route	Route Flow	Generalized Cost	Extended Route	Route Flow	Generalized Cost
R1	0.00	89.9127	R7	0.00	89.9402	R13	0.00	56.7126
R2	14.01	88.6829	R8	31.56	88.6829	R14	8.66	55.9623
R3	7.67	88.6248	R9	7.71	88.6248	R15	0.00	55.4247
R4	0.00	89.6232	R10	0.00	89.6099	R16	8.27	55.9623
R5	15.35	88.6248	R11	15.43	88.6248	R17	14.64	54.1837
R6	0.00	91.2948	R12	0.00	91.2782	R18	1.90	55.9623
R19	7.60	88.6247				R21	12.24	54.1837
R20	0.67	88.6829				R22	4.29	55.9623

**Table 3:** The change of the average VOT of each class against λ.

	λ										
	1.00	1.05	1.10	1.15	1.20	1.25	1.30	1.35	1.40	1.45	1.50
$\beta_{L}$	2.73	2.87	3.02	3.17	3.34	3.51	3.69	3.87	4.06	4.23	4.38
$\beta_{H}$	-	-	-	-	-	-	-	5.16	5.19	5.22	5.27
	λ										
	1.55	1.60	1.65	1.70	1.75	1.80	1.85	1.90	1.95	2.00	
$\beta_{L}$	4.51	4.61	4.68	4.74	4.78	4.81	4.84	-	-	-	
$\beta_{H}$	5.32	5.40	5.52	5.67	5.87	6.12	6.40	6.72	7.06	7.43	

**Table 4:** The path specification of the test network with HOT lane when *M* = 2.

OD Pair 1				OD Pair 2	
Path 1-3-2		Path 1-4-2		Path 3-2	
R1	(1-3${}^{L,\mathrm{SD}}$,3-2${}^{L,\mathrm{SD}}$)	R7	(1-4${}^{L,\mathrm{SD}}$,4-2${}^{L,\mathrm{SD}}$)	R13	(3-2${}^{L,\mathrm{SD}}$)
R2	(1-3${}^{H,\mathrm{SD}}$,3-2${}^{H,\mathrm{SD}}$)	R8	(1-4${}^{H,\mathrm{SD}}$,4-2${}^{H,\mathrm{SD}}$)	R14	(3-2${}^{H,\mathrm{SD}}$)
R3	(1-3${}^{L,\mathrm{RD}}$,3-2${}^{L,\mathrm{RD}}$)	R9	(1-4${}^{L,\mathrm{RD}}$,4-2${}^{L,\mathrm{RD}}$)	R15	(3-2${}^{L,\mathrm{RD}}$)
R4	(1-3${}^{H,\mathrm{RD}}$,3-2${}^{H,\mathrm{RD}}$)	R10	(1-4${}^{H,\mathrm{RD}}$,4-2${}^{H,\mathrm{RD}}$)	R16	(3-2${}^{H,\mathrm{SD}}$)
R5	(1-3${}^{L,\mathrm{RP}}$,3-2${}^{L,\mathrm{RP}}$)	R11	(1-4${}^{L,\mathrm{RP}}$,4-2${}^{L,\mathrm{RP}}$)	R17	(3-2${}^{L,\mathrm{RP}}$)
R6	(1-3${}^{H,\mathrm{RP}}$,3-2${}^{H,\mathrm{RP}}$)	R12	(1-4${}^{H,\mathrm{RP}}$,4-2${}^{H,\mathrm{RP}}$)	R18	(3-2${}^{H,\mathrm{RP}}$)
R19	(1-3${}^{L,\mathrm{SD}}$,3-2${}^{L,\mathrm{RD}}$)			R21	(3-2${}^{L,\mathrm{RP}\;*}$)
R20	(1-3${}^{H,\mathrm{SD}}$,3-2${}^{H,\mathrm{RD}}$)			R22	(3-2${}^{H,\mathrm{RP}\;*}$)
R23	(1-3${}^{L,\mathrm{SD}}$,3-2${}_{\mathrm{HOT}}^{L,\mathrm{SD}}$)			R29	(3-2${}_{\mathrm{HOT}}^{L,\mathrm{SD}}$)
R24	(1-3${}^{H,\mathrm{SD}}$,3-2${}_{\mathrm{HOT}}^{H,\mathrm{SD}}$)			R30	(3-2${}_{\mathrm{HOT}}^{H,\mathrm{SD}}$)
R25	(1-3${}^{L,\mathrm{RD}}$,3-2${}_{\mathrm{HOT}}^{L,\mathrm{RD}}$)			R31	(3-2${}_{\mathrm{HOT}}^{L,\mathrm{RD}}$)
R26	(1-3${}^{H,\mathrm{RD}}$,3-2${}_{\mathrm{HOT}}^{H,\mathrm{RD}}$)			R32	(3-2${}_{\mathrm{HOT}}^{H,\mathrm{RD}}$)
R27	(1-3${}^{L,\mathrm{RP}}$,3-2${}_{\mathrm{HOT}}^{L,\mathrm{RP}}$)			R33	(3-2${}_{\mathrm{HOT}}^{L,\mathrm{RP}}$)
R28	(1-3${}^{H,\mathrm{RP}}$,3-2${}_{\mathrm{HOT}}^{H,\mathrm{RP}}$)			R34	(3-2${}_{\mathrm{HOT}}^{H,\mathrm{RP}}$)
R35	(1-3${}^{L,\mathrm{SD}}$,3-2${}_{\mathrm{HOT}}^{L,\mathrm{RD}}$)			R37	(3-2${}_{\mathrm{HOT}}^{L,\mathrm{RP}\;*}$)
R36	(1-3${}^{H,\mathrm{SD}}$,3-2${}_{\mathrm{HOT}}^{H,\mathrm{RD}}$)			R38	(3-2${}_{\mathrm{HOT}}^{H,\mathrm{RP}\;*}$)

* Note that RPs on R21 and R22 can share rides with RDs on R19 and R20. Analogously, RDs on R37 and R38 can travel with RPs on R37 and R38.

## Abbreviations

The following abbreviations are used in this manuscript:

*N*	the set of nodes
*A*	the set of links, whose index is a∈A
*W*	the set of OD pairs, whose index is w∈W
$R_{w}$	the set of routs of OD pair w∈W
*R*	the set of routs of all OD pairs
$v_{a}^{m,i}$	number of travelers choosing mode *i* in class *m* on link *a* where i∈{SD,RD,RP}
*m*	a typical user class,m=1,2,···,M,*M* denotes the number of such traveler classes
$\delta_{ar}$	1, if link a∈A on path $r\in R_{w}$ and 0, otherwise
ϖ	vehicle capacity
$t_{a}$	travel time on link a∈A
$\lambda_{sr}^{+}$,$\lambda_{sr}^{-}$	multipliers for ride-matching constraints
$\rho^{m}$	a parameter for ridesharing passenger in class *m* to reflect the intolerance to congestion time
$\gamma^{\mathrm{RD}}$,$\gamma^{\mathrm{RP}}$	parameters of inconvenience cost for ridesharing driver and ridesharing passenger, respectively
$\omega^{\mathrm{RD}}$,$\omega^{\mathrm{RP}}$	parameters of compensation and payment for ridesharing driver and ridesharing passenger, respectively
$N_{rp}^{\mathrm{RP}}$	number of links where RDs travel with RPs
χ	guiding price for taking rides or for charging compensation
$I_{sr}^{\mathrm{RD}}$,$I_{rp}^{\mathrm{RP}}$	A simplified representation of the inconvenience cost functionsfor ridesharing driver and ridesharing passenger, respectively
$R_{sr}^{\mathrm{RD}}$,$R_{rp}^{\mathrm{RP}}$	A simplified representation of the compensation function and the payment function for RD and RP, respectively
$C_{r}^{m,\mathrm{SD}}$,${\tilde{C}}_{r}^{m,\mathrm{SD}}$	the normal travel cost function and the generalized cost function for solo driver in class *m* on path r
$C_{sr}^{m,\mathrm{RD}}$,${\tilde{C}}_{sr}^{m,\mathrm{RD}}$	the normal travel cost function and the generalized cost function for ridesharing driver in class *m* on path r who travels with ridesharing passenger on path *s*
$C_{rp}^{m,\mathrm{RP}}$,${\tilde{C}}_{rp}^{m,\mathrm{RP}}$	the normal travel cost function and the generalized cost function for ridesharing passenger in class *m* on path r who shares ride with ridesharing driver on path *p*
$D_{w}^{m}$	travel demand of class *m* between OD pair *w*
$\pi_{w}^{m}$	the minimum generalized path cost for traveler in class *m* between OD pair *w*
$\beta^{m}$	the average VOT for travelers of class *m*
*n*	parameters related to driving cost
θ	price of fuel per unit
